# Role of Histone Methylation in Maintenance of Genome Integrity

**DOI:** 10.3390/genes12071000

**Published:** 2021-06-29

**Authors:** Arjamand Mushtaq, Ulfat Syed Mir, Clayton R. Hunt, Shruti Pandita, Wajahat W. Tantray, Audesh Bhat, Raj K. Pandita, Mohammad Altaf, Tej K. Pandita

**Affiliations:** 1 Chromatin and Epigenetics Lab, Department of Biotechnology, University of Kashmir, Srinagar 190006, Jammu and Kashmir, India; arjamand96@gmail.com (A.M.); ulfatmir.scholar@kashmiruniversity.net (U.S.M.); wajahattantraryt.scholar@kashmiruniversity.net (W.W.T.); 2 Houston Methodist Research Institute, Houston, TX 77030, USA; crhunt305@gmail.com (C.R.H.); raj.pandita@bcm.edu (R.K.P.); 3 Division of Hematology and Medical Oncology, Saint Louis University, St. Louis, MO 63110, USA; spandit1896@gmail.com; 4 Centre for Molecular Biology, Central University of Jammu, Bagla 181143, Jammu and Kashmir, India; audeshs2002@gmail.com; 5 Centre for Interdisciplinary Research and Innovations, University of Kashmir, Srinagar 190006, Jammu and Kashmir, India; 6 Baylor College of Medicine, One Baylor Plaza, Houston, TX 77030, USA

**Keywords:** histone methylation, DNA repair, homologous recombination, non-homologous end joining

## Abstract

Packaging of the eukaryotic genome with histone and other proteins forms a chromatin structure that regulates the outcome of all DNA mediated processes. The cellular pathways that ensure genomic stability detect and repair DNA damage through mechanisms that are critically dependent upon chromatin structures established by histones and, particularly upon transient histone post-translational modifications. Though subjected to a range of modifications, histone methylation is especially crucial for DNA damage repair, as the methylated histones often form platforms for subsequent repair protein binding at damaged sites. In this review, we highlight and discuss how histone methylation impacts the maintenance of genome integrity through effects related to DNA repair and repair pathway choice.

## 1. Introduction

The packaging of eukaryotic DNA with histone proteins forms the fundamental unit of chromatin called the nucleosome. Nucleosomes contains 146 base pairs of DNA wrapped around a histone octamer containing two each of the H2A, H2B, H3, and H4 histones [1,2]. Apart from histone, non-histone proteins also bind to DNA and alter the chromatin structure. The compaction of large DNA segments into chromatin imposes a barrier to proteins that need access to a DNA template for processes such as transcription, replication, recombination, and repair [2]. Chromatin structure can be modulated by various mechanisms, including ATP-dependent chromatin remodeling proteins, histone variant exchange, and histone post-translational modifications, to ensure access by various proteins to the DNA [2,3]. The eukaryotic genome is constantly challenged by various exogenous and endogenous DNA damaging agents, such as reactive oxygen intermediates, UV light, ionizing radiation, and other chemical agents that cause various types of DNA breaks [4]. Accurate repair of damaged DNA is essential for genomic stability. Failure to repair DNA breaks can lead to various diseases including cancer, ageing, and neurodegenerative disorders [4,5]. Therefore, it is important that cells identify the breaks and initiate and activate processes to repair the damaged DNA [6,7]. Cells respond to breaks in the genome by activating a network of pathways, collectively called the DNA damage response (DDR), that detects breaks and generates an appropriate protective response [8]. One of the most deleterious types of DNA damage is DNA double-strand break (DSB) [9,10,11,12]. Two important pathways exist to repair DSBs: non-homologous end-joining (NHEJ) and homologous recombination (HR) [13]. NHEJ utilizes an error-prone, direct repair mechanism, and is active in the G0 and G1 phases of the cell cycle. In contrast, HR uses a homologous DNA sequence as a repair template, is highly accurate, and is mostly active in the S and G2 phases of the cell cycle [14,15]. Chromatin-based mechanisms play a crucial role in DNA damage repair by marking the damage sites and initiating signaling cascades to coordinate repair processes. 

**Figure 1:**
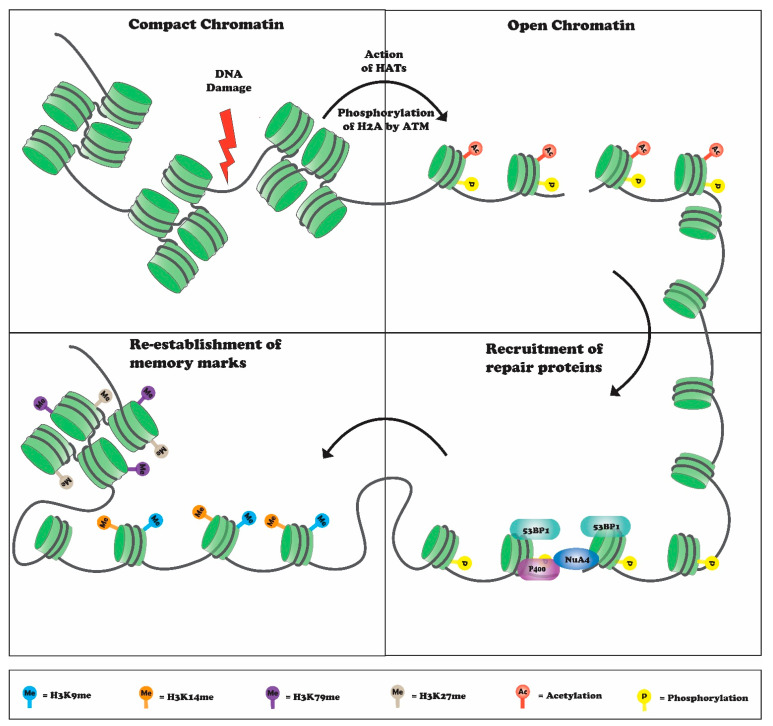
DNA damage initiates chromatin decompaction mediated by various histone acetyltransferases (HATs) and recruits various DSB-repair and chromatin-modifying proteins. Binding of these proteins, such as 53BP1, NuA4, and P400, leads to DSB repair followed by chromatin compaction, which helps in maintaining genome integrity.

Histone proteins undergo various post-translational modifications (PTMs), such as phosphorylation, acetylation, methylation, ubiquitylation, and sumoylation [1,2]. These modifications influence chromatin structure by altering histone DNA interactions, and by acting as docking sites for various proteins to regulate essential aspects of DNA-dependent transactions [1,2]. In response to DNA damage, histone modifications are critical for DNA break repair and cell survival [16]. Histone modifications help to sense DNA damage, facilitate recruitment of repair factors to the break site, and re-establish a normal chromatin structure after repair (Figure 1) [16]. An especially prominent and widely studied modification with respect to transcription and DNA repair is histone methylation. Additional cellular processes regulated by histone methylation include X-chromosome inactivation, cell differentiation, and heterochromatin formation [17]. Accumulating evidence suggests that histone methylation is important for the repair of DSBs and contributes to repair pathway choice [18]. Several lysine residues in histones are modified in response to DNA damage, these include histone H3K4, H3K9, H3K27, H3K36, H3K79, and histone H4 lysine 20. In this review, we focus on how histone methylations regulate the DNA damage response.

## 2. Histone H4K20 Methylation in DNA Repair

**Figure 2:**
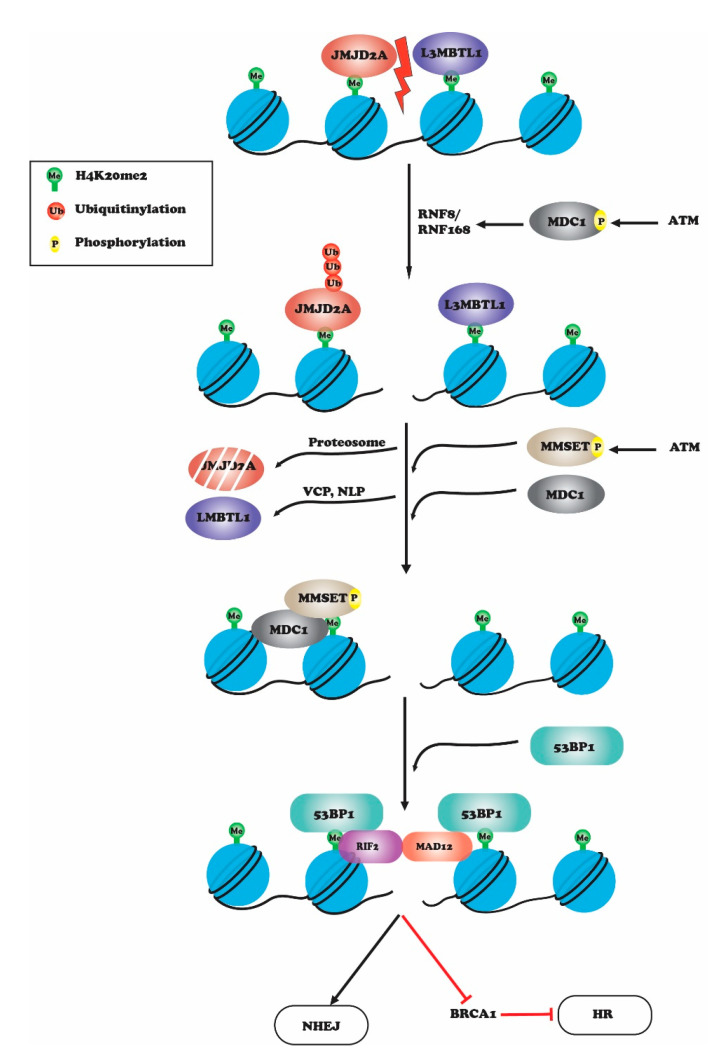
Upon DNA damage, L3MBTL1 and JMJD2A are released from H4K20me2 through ATM-mediated recruitment of MDC1 and phosphorylation of MDC1 at Ser 139. This leads to accumulation of RNF8 and RNF168 at DSBs, which causes ubiquitinoylation and degradation of JMJD2A by proteosome-mediated degradation and removal of L3MBTL1 by ATPase valosin-containing protein (VCP) and nuclear protein localizing cofactor protein 4 (NPL4). Thus, upon DNA damage, exposed H4K20me2 becomes available for 53BP1 binding to initiate NHEJ. 53BP1 recruits downstream effector proteins RIF1 and MAD12, which inhibit BRCA1 protein-binding to promote NHEJ over HR in the G1 phase of the cell cycle.

Histone H4 lysine 20 methylation is the only reported methylation site on H4 that has a role in maintaining genome integrity upon DNA damage [19]. H4K20 methylation is catalyzed by several histone methyltransferases: PR-Set7/Set8/KMT5A is responsible for monomethylation of H4K20, while H4K20me2/3 methylation is catalyzed by SUV4-20h1/2 [19]. H4K20 methylation levels do not change upon DNA damage, but the preexisting H4K20me becomes exposed and assists repair protein recruitment to the damage site [20,21]. H4K20 methylation can be regulated by Epidermal Growth Factor Receptor (EGFR), which phosphorylates H4Y72 and leads to increased H4K20 methylation levels [22] by increasing the interaction of histone H4 with Set8 and Suv420H methyltransferase [22]. A H4Y72F mutant displays reduced DNA repair activity upon IR induced DNA damage [22]. H4K20 methylation plays a prominent role in NHEJ by serving as a binding site for 53BP1 at damage sites, which then stimulates a downstream cascade involving DSB responsive proteins and checkpoint signaling proteins [23,24]; 53BP1 binds to methylated H4K20 via its tandem tudor domain [25]. In fission yeast, Set9-meditated methylation of H4K20 localizes Crb2 (53BP1 ortholog) to DNA damage sites [21,26]. In normal cells, methyl-binding proteins, L3MBTL1 (lethal [3] malignant brain tumor like protein 1) and JMJD2A/KDM4A bind to H4K20me2 and thus prevent binding of 53BP1 in the absence of DNA damage [27,28,29,30,31]. Binding of 53BP1 to H4K20-methylated chromatin is also obstructed by neighboring H4K16 acetylation. Conversely, deacetylation of H4K16 increases binding of 53BP1-H4K20me2 at DSB sites [32]. L3MBTL1 and JMJD2A are released from H4K20me2 upon induction of DNA damage through ATM-mediated recruitment of MDC1 (mediator of DNA damage checkpoint 1) and phosphorylation of MDC1 at Ser 139. This leads to an accumulation of RNF8 and RNF168 at DSBs that causes ubiquitinoylation of L3MBTL1 and JMJD2A [33,34,35]. The removal of ubiquitinylated L3MDTL1 is mediated by ATPase valosin-containing protein (VCP) and nuclear protein localizing cofactor protein 4 (NPL4), while ubiquitinylated JMJD2A undergoes proteosome-mediated degradation [33]. Thus, upon DNA damage, exposed H4K20me2 becomes available for 53BP1 binding to initiate NHEJ [34,35]; 53BP1 recruits downstream effector proteins RIF1 and MAD12, which inhibit BRCA1 protein-binding to promote NHEJ over HR in the G1 phase of the cell cycle (Figure 2) [36,37,38]. In response to DNA damage, 53BP1 and BRCA1 competition regulates repair pathway choice between NHEJ and HR. Cells lacking BRCA1 have impaired HR repair and BRCA1-deficient mice are embryonically lethal, a phenotype that can be rescued by loss of 53BP1. These findings indicate that in absence of BRCA1, 53BP1 blocks HR repair [39,40,41]. The data also suggest that proper regulation of pathway choice is critical for the maintenance of genome stability and that H4K20 methylation plays an essential role in deciding the repair pathway choice. In response to DNA damage, TIP60-mediated acetylation of H4K16 and H2AK15 block 53BP1 binding and favor HR. Acetylated H4K16 prevents 53BP1 binding to the H4K20-methylated residue due to steric hindrance [42]. Acetylation of H2AK15 by TIP60 prevents its ubiquitylation, which is also a recognition site for 53BP1 [20,25,43]. 

SET8-dependent monomethylation of histone H4K20 has also been shown to play an important role in chromatin organization upon cell exit from mitosis [44,45,46,47,48]. Depletion of SET8 or mutation of H4K20 residues leads to genome-wide chromatin decompaction in daughter cells, which results in an excessive loading of origin recognition complex (ORC) in daughter cells [49]. ORC loading causes aberrant MCF7 helicase recruitment to chromatin, causing single-stranded DNA formation and DNA damage [44,49,50]. Further, single-stranded binding protein, RPA, levels are elevated in SET8 deleted cells, while cells lacking both SET8 and MCF7 show decreased ssDNA levels [44]. SET8 is required for chromatin compaction during the cell-cycle transition from M to G1 phases. Deletion of SET8 or mutation of H4K20 to H4K20A/R increases γH2AX levels [44]. Similarly, treatment with the SET-8 inhibitor UNC0379 decreases H4K20me1 levels and leads to developmental arrest at the one-celled stage [51]. These studies suggest that multiple proteins and critical histone modifications regulate the DNA damage response through impacts on H4K20 methylation.

## 3. Histone H3K4 Methylation in DNA Repair

Methylation of histone H3 at lysine 4 by Set1p histone methyltransferase is associated with transcriptional activation and a proper response to DNA damage. Ubiquitination of H2BK123 by Rad6/Bre1 is a prerequisite for methylation of H3K4 [52,53,54]. In budding yeast, cells lacking Set1, or with a mutation in H3K4, have significantly compromised DSB repair by the NHEJ pathway and decreased survival in the presence of replication stress [55]. Set1 binding and H3K4me3 levels are enriched around DSB break sites. H3K4 methylation accumulates at the homothallic switching endonuclease (HO)-induced break site in budding yeast or at breaks induced by I-SceI endonuclease in mammalian cells [56]. Set1 recruitment to DNA break sites is dependent on the RSC complex chromatin remodeler. Defects in H3K4 methylation or RSC depletion impairs DSB repair by the NHEJ pathway and these cells also display defects in S-phase transition during replication stress [56,57,58]. While induction of DNA damage by phleomycin or neocarzinostatin (NCS) does not increase global levels of H3K4 methylation, there is localized increase in H3K4me3 at the break sites, suggesting trimethylation of H3K4 contributes to DNA damage signaling [56,59,60]. Inducible H3K4me is not involved in transcription, which is repressed around the break sites. RSC-dependent H3K4me3 is speculated to be vital for opening of the chromatin at the break site [56]. During transcription, H3K4me2/3 recruits chromatin remodeling ATPase hSNF2H in humans and Isw1p in yeast [61]. In addition, H3K4me3 helps in the recruitment of SNF2H at the DNA damage sites [62]. Further, H3K4me3 provides a binding site for inhibitor of growth (ING1) to stimulate DNA damage repair post UV irradiation and promote damage-induced apoptosis [63,64]. Thus, H3K4me3 acts as a platform for various proteins involved in DDR. It has also been observed that H3K4 demethylation by KDM5B at damage sites is important for the repair of DNA lesions in human cells [65,66]. Demethylation is thought to modulate the chromatin structure from a transcriptionally favored state to a chromatin state that facilitates DNA repair. KDM5B was reported to be enriched at I-SceI- induced DSB sites in a PARP1- and macroH2A1.1-dependent manner [67]. Furthermore, catalytically dead mutations of KDM5B, or KDM5B loss, abolish BRCA1 and Ku70 recruitment to damage sites and leads to defective HR and NHEJ repair [67]. Another demethylase, KDM5A, demethylates H3K4me3 and facilitates the recruitment of the chromatin remodeling complex ZMYND8-NuRD to DNA damage sites [68]. ZMYND8-NuRD represses transcription around the DNA double-strand break site. Cells lacking KDM5A manifest impaired transcriptional repression and HR repair at DSBs similar to that observed after ZMYND8-NuRD loss [18]. In addition to KDM5A and KDM5B, the KDM5C H3K4me3 demethylase plays a role in the DDR in response to replication stress induced by alkylating agents such as methyl methanesulfonate (MMS). After simulation, KDM5C is recruited to chromatin, where it demethylates H3K4me3 in order to maintain the repressed chromatin state [69]. Thus, different demethylases play different roles depending upon the nature of the DNA damage.

## 4. H3K36 Methylation in the DNA Damage Response

**Figure 3:**
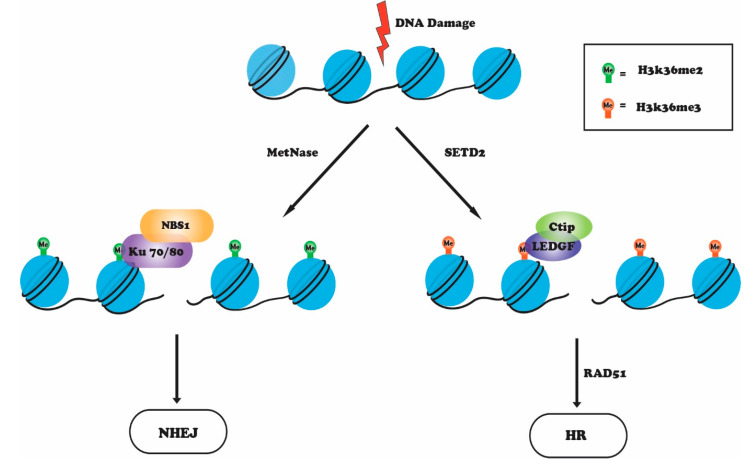
Upon DNA damage, metnase catalyzes H3K36me2, which recruits Ku70-NBS1 complex, to repair DNA damage via NHEJ pathway, whereas H3K36me3 catalyzed by SETD2 favors the HR pathway by recruiting LEDGF-CtIP complex. This is followed by RAD51 recruitment and, in turn, HR repair.

The methylation of histone H3 at lysine 36 (H3K36) is catalyzed by the Set2 histone methyltransferase. Set2 in *Saccharomyces cerevisiae* is responsible for all forms of H3K36 methylation (mono-, di-, and tri-) [70,71]. In humans, several enzymes can methylate H3K36, but SETD2 (KMT3A) is the only methyltransferase that trimethylates H3K36 [18,72]. Methylated H3K36 is highly enriched within the coding regions of actively transcribed genes through the association of SETD2 with the C-terminal domain (CTD) of RNA Pol ll as part of the transcription elongation machinery [70]. H3K36 methylation is also involved in splicing and suppression of cryptic intragenic transcription [72,73]. In yeast, methylated H3K36 recruits RPD3 histone deacetylase complex to chromatin, which maintains a repressive state. This prevents aberrant transcription initiation from cryptic sites and histone exchange in transcribed regions by regulating the activity of Asf1, Chd1, and ISW1b complexes [74,75,76]. In budding yeast, loss of Set2 leads to hypersensitivity to DNA damaging agents and site-specific double-strand breaks. Furthermore, these cells fail to activate checkpoint signaling, show impaired response to DNA damage, and inappropriate DNA break-site resection in G1 phase cells [77]. Several reports in mammals have linked H3K36 methylation with the DNA damage response that occurs preferentially at breaks in transcriptionally active regions of the genome [78]. Depletion of SETD2 leads to decreased phosphorylation of ATM and p53, defective DNA end resection and a loss of recruitment of RPA and RAD51 to damaged sites, and reduced HR efficiency [78,79,80]. H3K36 methylation is important for HR repair as it acts as a docking site for the PWWP methyl binding domain of lens epithelium derived growth factor (LEDGF) [81]. Upon DNA damage, LEDGF binding to H3K36me3 enables the recruitment of C-terminal binding protein interacting protein (CtIP), a DNA damage response factor, to DNA DSB sites and promotes the CtIP-dependent resection steps associated with DSB repair by HR [81]. Depletion of SETD2 impairs LEDGF binding to chromatin, which hinders CtIP recruitment, resulting in defective end-resection and a reduction in ssDNA binding proteins RPA and RAD51 at the damage sites [78,81,82]. Overexpression of H3K36me3 demethylase KDM4A (JMJD2A or JHDM3A) decreases HR efficiency [79]. In contrast with H3K36 trimethylation, which favors HR, dimethylation of H3K36 promotes NHEJ [83,84]. IR-induced DSBs cause enrichment of H3K36me2 around the break sites and binding of NHEJ proteins. Metnase (SETMAR) is recruited to damage sites and mediates demethylation of H3K36 around the break site [85,86]. Dimethylated H3K36 leads to recruitment and stabilization of Ku70/Ku80, PHD and ring finger domain 1 (PHRF1), and NBS1, thereby promoting DSB repair by the NHEJ pathway [87]. Depletion of metnase or H3K36me2 depletion by demethylase KDM2A knockdown inhibits the NHEJ repair pathway. Furthermore, mutation of H3K36 to H3R36 or H3A36 results in a marked decrease in the recruitment of Ku70 and NBS1 to DSBs, indicating that H3K36me2 serves as a docking site for the assembly of repair proteins at DSBs and for efficient DSB repair [83,87]. Although these findings indicate that H3K36me2 and H3K36me3 methylation assist DSBs repair by either HR or NHEJ (Figure 3), the precise molecular factors that govern the activation of these repair pathways are not fully known.

## 5. Histone H3K79 Methylation in DNA Repair

Unlike most histone methylations that occur on histone tails, H3K79 methylation occurs within the globular domain of histone H3 [1,88]. H3K79 is methylated by an evolutionarily conserved non-SET containing histone methyltransferase called disruptor of telomeric silencing-1 (hDot1). Dot1 was initially discovered as a gene whose overexpression causes silencing defects at telomeres in budding yeast. Dot1 methylates H3K79 in a nucleosomal context and requires an H4 N-terminal tail for binding and subsequent methylation of H3K79 [1,89]. Ubiquitination of histone H2B at lysine K123 is a prerequisite for H3K79 methylation [90]. H3K79 methylation plays important roles in transcription, telomeric silencing, and cell-cycle regulation [1,91]. Studies across multiple species have linked Dot1-mediated H3K79 methylation with DNA-damage signaling, with H3K79 methylation shown to act as a binding site for 53BP1 repair protein in humans, and its ortholog Rad9 in yeast, to DNA damage sites. Both 53BP1 and Rad9 bind to methylated H3K79 chromatin through their tudor domains. Depletion of Dot1 or mutation of H3K79 impairs recruitment of 53BP1 or Rad9 to DNA DSB sites [92]. Similarly, mutations in the tudor domain of 53BP1 or Rad9 abolish their recruitment to DSBs [93,94]. There are several reports demonstrating that binding of 53BP1 to chromatin depends on H4K20 methylation. 53BP1 recognizes H4K20 methylation through its tudor domain as mutations in the tudor domain abolish 53BP1 recruitment. It appears that the choice of 53BP1 binding to methylated H3K79 or H4K20 is regulated by the cell-cycle phase. During the G1 and G2 phases of the cell cycle, when H4K20 levels are low, 53BP1 binds to H3K79, while in the S phase, when H3K79 methylation is low, H4K20 methylation is required for 53BP1 foci formation in response to DNA damage [95]. In budding yeast, recruitment of Rad9 by H3K79me3 is important to G2 phase DNA damage repair because it limits ssDNA production during non-homologous end joining [96]. Budding yeast lacking Dot1 or with mutated H3K79 shows IR sensitivity and leads to defective G1–S phase checkpoint activation. In addition to checkpoint activation, Dot1 is involved in homologous recombination through cohesion loading [97]. H3K79 methylation was also shown to be critical for nucleotide excision repair (NER) in response to UV-induced DNA damage, as cells lacking Dot1 or with mutated H3K79 are UV hypersensitive [98]. H3K79 methylation might help in recruiting XPC, which in turn enhances efficient removal of UV photoproducts. DNA damage induced by UV irradiation causes a blockade of transcription, and Dot1 was shown to be required for transcriptional restart after nucleotide excision repair. Cells lacking Dot1 show impaired transcription restart [99]. Dot1-mediated H3K79 methylation thus plays an important role in the repair of damaged DNA at various levels.

## 6. Crosstalk between H3K9 Methylation, ATM and TIP60

**Figure 4:**
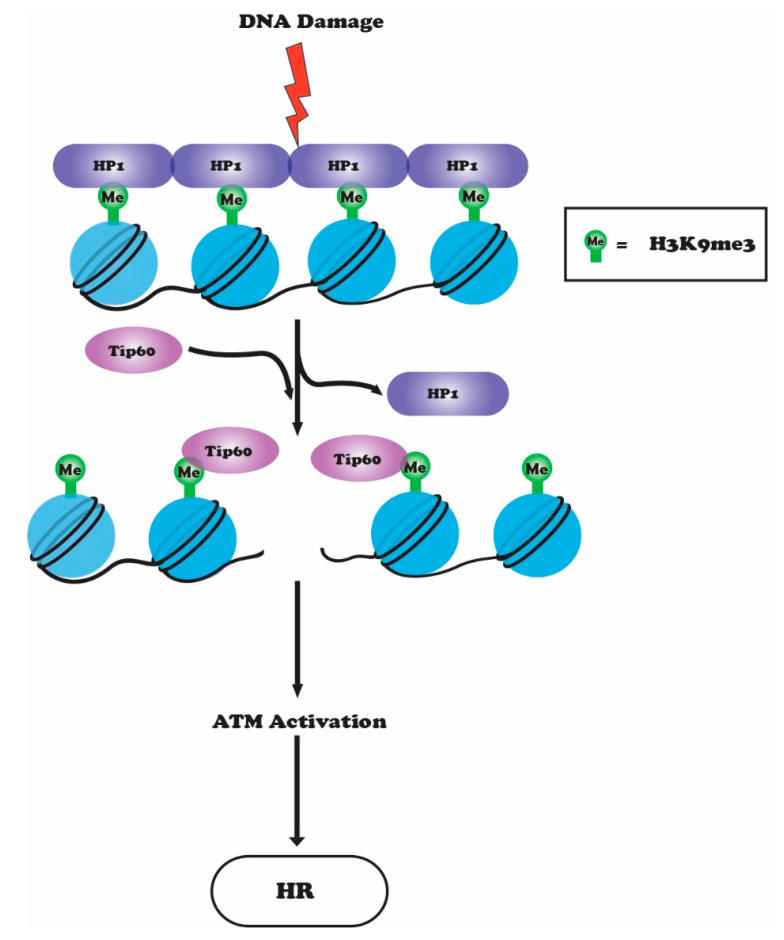
In response to DNA damage, HP1 is displaced from the H3K9me3 site allowing TIP60 to interact with H3K9me3 via its chromodomain. The interaction of TIP60 with H3K9me3 stimulates its HAT activity, which leads to acetylation of ATM and subsequent activation of its kinase activity, downstream ATM signaling, and HR-mediated repair.

Histone H3 methylation at lysine 9 (H3K9me3) is mainly associated with heterochromatin-mediated gene silencing [100,101]. H3K9 methylation is catalyzed by histone methyltransferase suppressor of variegation 3–9 homolog 1 (Suv39H1) or its homolog Suv39H2 [102,103,104]. In humans, there are eight H3K9 histone methyltransferases (SUV39h1, SUV39h2, G9a, SETDB1, SETDB2, PRDM2, PRDM3, and PRDM16) that show considerable functional redundancy [105,106]. *C. elegans* has two H3K9-specific methyltransferases, MET-2 (SETDB1 homolog) and SET-25 (G9a/ SUV39H1 related). MET-2 is responsible for H3K9me1 and H3K9me2, while SET-25 catalyzes the final trimethylation step of H3K9 [107]. MET-2-mediated K3K9me2 occurs at satellite simple repeat sequences and is responsible for transcriptional repression. Depletion of MET-2 leads to an accumulation in satellite repeat transcripts and a loss of BRCA1/BARD1, which leads to RNA:DNA (R loop) hybrid formation on repetitive sequences [107,108,109]. SET-25-dependent H3K9me3 is mainly associated with the repression of transposable elements and silent tissue-specific genes [107]. Silencing of tandem repeats and transposable elements are important for maintenance of genome integrity and any perturbation would lead to genome instability [105]. Double mutants of Met-2 and Set-25, which lack all forms of H3K9 methylation, are sterile with extensive DNA damage in germ line cells. Furthermore, in double mutants, transposons and simple repeats are de-repressed in both germline and somatic tissues [109]. In humans, recruitment of Suv39H1 and Suv39H2 to DNA DSBs increases H3K9 methylation around the break sites [110]. H3K9 methylation sites are then recognized by the histone acetyltransferase TIP60 through its chromodomain [104]. The interaction of TIP60 with H3K9me3 stimulates its HAT activity, which increases acetylation of H4, H2A, and ATM, and its increased kinase activity subsequently initiates downstream ATM signaling and HR repair [42,104]. Methylation of H3K9 also helps to increase the binding of histone methyltransferase SUV39H1, KAP1, and HP1 complex to DSBs, which further help in spreading of H3K9 methylation, more TIP60 recruitment, and additional TIP60-mediated ATM activation [103]. ATM activation ultimately releases SUV39H1-KAP1-HP1 complex from the break sites by phosphorylating KAP1 [103]. Acetylation of H4 and H2A by TIP60 around break sites prevents 53BP1 binding, which would favor NHEJ repair by preventing DNA end-resection [33,42]. Thus, TIP60 promotes HR by preventing 53BP1 binding (Figure 4). Depletion of SUV39H1 or H3K9 mutation decreases TIP60-mediated histone acetylation around DSB sites and hence impairs HR repair [103]. Histone demethylases KDM4B (JMJD2B) and KDM4D (JMJD2D) specific to H3K9me3 have also been shown to play a role in DDR [111,112,113]. PARP1, a poly ADP-ribose polymerase, recruits these KDMs to the DNA damage sites. Upon DNA damage, KDM4D is PARylated by PARP1, and depletion of KDM4D impairs association of ATM with chromatin and inhibits ATM-dependent signaling and phosphorylation of H2AX, KAP1, and CHK2 [111]. Cells depleted of KDM4D show reduced binding of Rad51 and 53BP1, and defects in both the HR and NHEJ pathways. The role of H3K9me3 demethylases in DNA repair is further supported by the finding that catalytically dead KDM4D mutant cells have HR defects similar to those in cells lacking KDM4D. However, the mechanism coordinating damage-induced H3K9me3 demethylation with H3K9 methyltransferases is not clear, nor is the precise mechanism by which the same residue can regulate pathway choice [18,111].

## 7. Conclusions and Future Perspectives

As part of the cellular response to DNA damage, a wide range of histone PTMs (phosphorylation, ubiquitylation, acetylation, and methylation) have been shown to play important roles in generating and regulating DDRs. In this review, we discussed the role of specific histone methylation sites ad enzymes in DNA DSB repair, and how they regulate pathway choice. Many histone methyltransferases and demethylases are recruited to chromatin in response to damage and change the local chromatin structure to facilitate repair-protein recruitment. However, there are still several gaps that need to be addressed to fully understand the role of histone methylation in DNA repair. Several histone methyltransferases and demethyltransferases target the same histone site, how is the activity of these enzymes regulated or their targeting to the same genomic loci? Sometimes the same modification can regulate both HR and NHEJ depending on the level of modification, so knowing the whole repertoire of methyl readers would shed light on how these modifications regulate different repair pathways. It has also been shown that both histone methyltransferases and demethylases against specific methylations play a role in DNA repair, but how the activities of these two opposing enzymes regulate the same outcome in the context of DNA repair needs to be elucidated further. Mutations of various methylation sites, or misregulation of methyltransferases or demethylases, and the related failure to repair damaged DNA in various diseases need to be further analyzed. The nature of the chromatin state before and after DNA damage, how that structure varies between different genomic loci, and the influence of specific types of DNA damage on repair outcomes are all challenging questions that need to be addressed.

## Data Availability

Not Applicable.
